# When Back Pain Is Cardiac: Spontaneous Coronary Artery Dissection in a Male Patient

**DOI:** 10.7759/cureus.103055

**Published:** 2026-02-05

**Authors:** Mohammed Martini, Hajar El Amri, Yara Deeb, Rami Al Ayyubi, Abhishek Kulkarni

**Affiliations:** 1 Internal Medicine, Southern Illinois University School of Medicine, Springfield, USA; 2 Cardiology, Southern Illinois University School of Medicine, Springfield, USA

**Keywords:** : acute coronary syndrome, atypical acute coronary syndrome, atypical spontaneous coronary artery dissection, male patient, myocardial infarction with non-obstructive coronary arteries (minoca), non atherosclerotic myocardial infarction, non-st segment elevation myocardial infarction (nstemi), spontaneous coronary dissection, type ii spontaneous coronary artery dissection

## Abstract

Spontaneous coronary artery dissection (SCAD) is a rare, non-atherosclerotic cause of acute coronary syndrome that is more commonly described in women and is frequently underrecognized in men, particularly when presenting with atypical symptoms. We report the case of a 48-year-old man with a history of hypertension and diabetes who presented with intermittent infrascapular back pain for two weeks without typical anginal features. Initial evaluation revealed markedly elevated cardiac troponin levels, while electrocardiography showed no ischemic changes. Transthoracic echocardiography demonstrated preserved left ventricular systolic function with new regional wall motion abnormalities. Coronary angiography revealed a discrete lesion with diffuse distal narrowing of the first obtuse marginal artery, consistent with a Type 2 spontaneous coronary artery dissection. Given the patient’s hemodynamic stability and absence of ongoing ischemia, a conservative management strategy was pursued with antiplatelet therapy and beta-blockade. The patient’s symptoms resolved during hospitalization, and he was discharged in stable condition with close outpatient follow-up. This case highlights the diagnostic challenges of SCAD in male patients with atypical presentations and underscores the importance of maintaining a high index of suspicion to ensure timely diagnosis. It also supports conservative medical management as a safe and effective approach in stable patients with SCAD and emphasizes the need for increased awareness and recognition of this condition in populations where it is less commonly reported.

## Introduction

Spontaneous coronary artery dissection (SCAD) is an uncommon, non-atherosclerotic cause of acute coronary syndrome (ACS) and myocardial infarction in patients without obstructive coronary artery disease. It is estimated to account for approximately 1-4% of all ACS presentations and up to 35% of myocardial infarctions in younger patients without traditional cardiovascular disease risk factors [1,2]. Despite increasing recognition over the past decade, SCAD remains underdiagnosed due to its heterogeneous clinical presentation and often subtle angiographic features.

SCAD predominantly affects women, particularly those who are young or middle-aged, with approximately 90% of reported cases occurring in females [3]. In contrast, SCAD in men is relatively rare and is more commonly associated with intense physical exertion or isometric stress. As a result, the diagnosis may be overlooked in male patients, especially when presenting symptoms are atypical and not consistent with classic anginal chest pain.

Early and accurate identification of SCAD is essential, as management strategies differ substantially from those used for atherosclerotic ACS. Percutaneous coronary intervention carries higher complication rates in SCAD, and conservative medical therapy is preferred in clinically stable patients [1]. We present a case of spontaneous coronary artery dissection in a middle-aged male presenting with infrascapular back pain, initially raising diagnostic uncertainty. This case highlights the importance of maintaining a high index of suspicion for SCAD in men with atypical symptoms and underscores the value of recognizing characteristic angiographic patterns to guide appropriate management.

## Case presentation

A 48-year-old male with a history of type 2 diabetes with hemoglobin A1c 8.8% on Tirzepatide, hypertension on treatment with Lisinopril and anxiety, presented with intermittent left infrascapular pain that had been ongoing for two weeks. The pain was non-radiating, episodic, and not clearly associated with exertion. He denied accompanying symptoms such as dyspnea, dyspepsia, or palpitations at the time of presentation. Notably, one year prior, he’d experienced exertional chest pain and undergone coronary angiography, which revealed minimal non-obstructive coronary artery disease.

On presentation, his vital signs were notable for elevated blood pressure (147/111 mmHg) with a heart rate of 84 beats per minute. Laboratory workup revealed elevated troponin levels with a rising trend, as summarized in Table 1.

**Table 1:** Serial cardiac troponin measurements demonstrating myocardial injury

Time from presentation	Cardiac troponin (ng/L)	Reference range (ng/L)
Initial	3319	<34
6 hours (peak)	11741	<34

**Figure 1:**
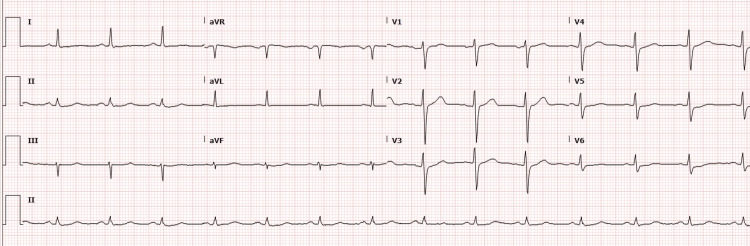
Twelve-lead electrocardiogram showing normal sinus rhythm without acute ischemic ST-T changes.

**Figure 2:**
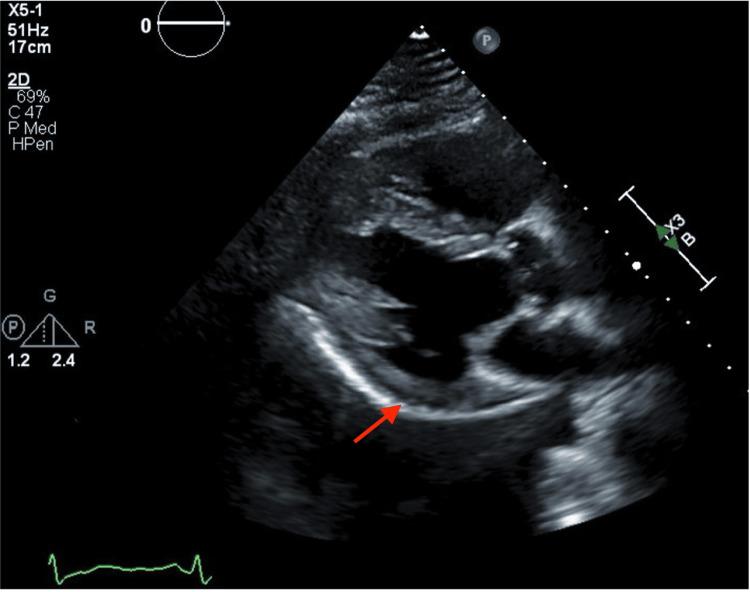
Parasternal long-axis echocardiographic view demonstrating inferolateral left ventricular hypokinesis, correlating with ischemia in the affected myocardial territory

The electrocardiogram showed a normal sinus rhythm without ST-T segment abnormalities (Figure 1). Given concern for non-ST-elevation myocardial infarction (NSTEMI), the patient was loaded with aspirin and started on atorvastatin and a heparin infusion. Transthoracic echocardiography demonstrated a preserved left ventricular ejection fraction of 57% with new regional wall motion abnormalities involving the basal and mid anterolateral and basal inferolateral segments (Figure 2).

**Figure 3:**
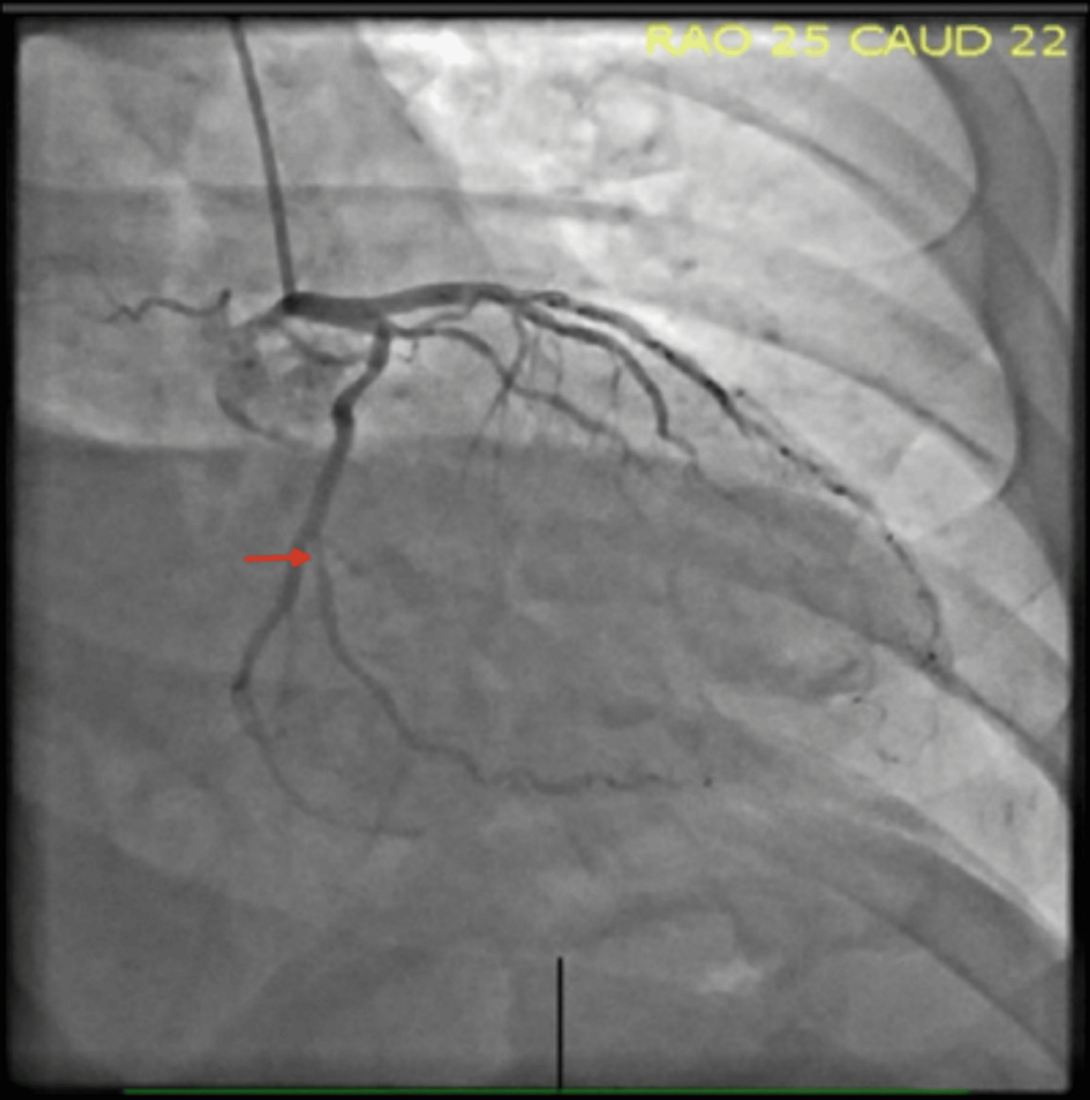
Coronary angiography demonstrating Type 2 spontaneous coronary artery dissection of the first obtuse marginal artery. Right anterior oblique (RAO) caudal view showing a discrete proximal narrowing of the first obtuse marginal artery (red arrow)

**Figure 4:**
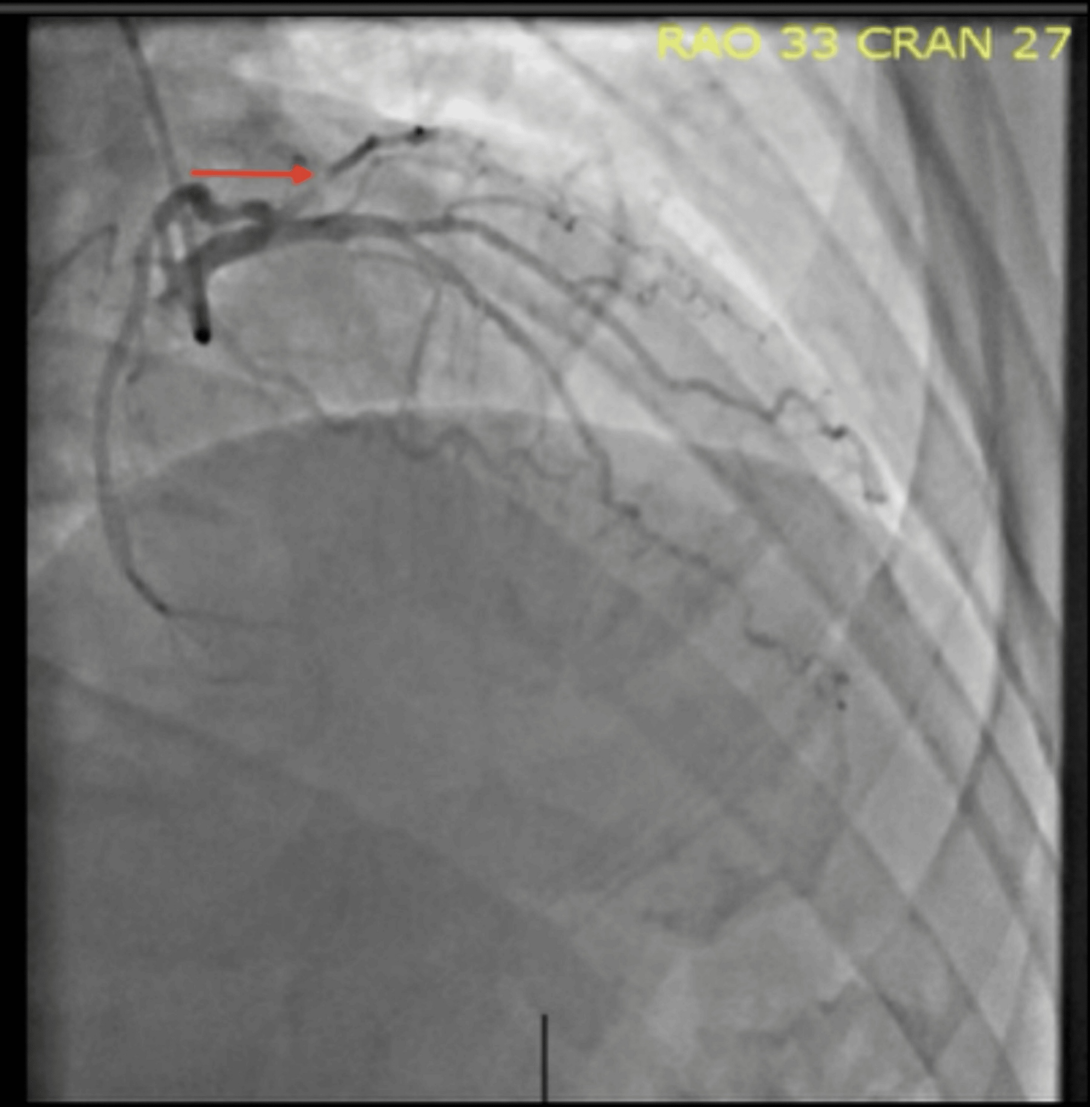
Coronary angiography with RAO cranial view demonstrating persistent diffuse narrowing of the same vessel segment, further supporting the diagnosis of Type 2 spontaneous coronary artery dissection (red arrow).

Subsequent coronary angiography revealed a discrete lesion in the first obtuse marginal artery, followed by diffuse narrowing distal to the lesion, findings consistent with a SCAD (Figures 3, 4). Given the patient’s stable condition, a conservative management strategy was pursued. He was started on antiplatelet therapy with aspirin and a beta-blocker, with strict recommendations to avoid heavy physical exertion. His symptoms resolved during hospitalization, and he was discharged in stable condition with no active pain and plans for close outpatient follow-up

## Discussion

SCAD is a nonatherosclerotic, nontraumatic dissection of the coronary artery wall. It accounts for approximately 1%-4% of ACS cases; however, its true prevalence is likely underestimated due to diagnostic challenges and frequent underrecognition [1]. SCAD is being increasingly recognized as a subtype of myocardial infarction in the absence of obstructive coronary artery disease (MINOCA), especially with the increased use of intracoronary imaging techniques; the disease itself is more recognized among younger women [2]. MINOCA is a syndrome that accounts for approximately 6-8% of all myocardial infarctions [2]. Differentiating SCAD from other etiologies of MINOCA is critical, as therapeutic approaches and long-term management strategies differ significantly.

The exact pathogenesis of SCAD remains incompletely understood. Two primary mechanisms have been proposed: one involves an intimal tear that allows blood to enter and form a false lumen within the vessel wall; the other suggests a spontaneous hemorrhage from the vasa vasorum, leading to intramural hematoma and subsequent vessel obstruction [1].

SCAD affects women much more frequently than men; only 10% of cases occur in men, a disparity thought to be influenced by hormonal factors [3]. Elevated estrogen levels, particularly during pregnancy and the postpartum period, are believed to increase susceptibility. Additional associated factors include emotional stress and fibromuscular dysplasia. In contrast, SCAD in men has been more commonly linked to strenuous physical exertion, and usually patients will have traditional cardiovascular risk factors, including hypertension and diabetes [3].

The clinical symptoms of SCAD are predominantly typical of ACS, with chest pain being the most prevalent symptom in 85% to 95% of cases [4]. However, our patient presented atypically with left-sided back and shoulder pain, rather than classical cardiac chest pain, which can contribute to delays in recognition and diagnosis.

The left anterior descending (LAD) artery is the most affected vessel in both men and women. Involvement of the circumflex, ramus, and obtuse marginal branches, such as in our patient, occurs in approximately 15%-45% of cases [1].

Coronary angiography is the gold standard for diagnosing SCAD [5]. The Yip-Saw angiographic classification divides SCAD into three types: Type 1 is characterized by multiple radiolucent lumens; Type 2 shows long, diffuse, and smooth narrowing often indicative of intramural hematoma; Type 3 presents as focal or tubular stenosis, resembling atherosclerosis or thrombus [6].

In cases where the diagnosis is uncertain, intracoronary imaging modalities such as optical coherence tomography (OCT) or intravascular ultrasound (IVUS) can aid in confirmation. However, their use carries the potential risk of exacerbating the dissection or causing iatrogenic injury [6], such as contrast-induced hydraulic extension of the dissection during OCT imaging [2]

Management of spontaneous coronary artery dissection is primarily conservative in clinically stable patients. The role of dual antiplatelet therapy (DAPT) in conservatively managed SCAD remains uncertain, as comparative evidence between DAPT and aspirin monotherapy is lacking. While DAPT may theoretically reduce thrombus formation at the site of intimal disruption, this potential benefit must be weighed against an increased risk of bleeding and propagation of intramural hematoma [1]. Accordingly, antiplatelet therapy in SCAD should be individualized. In clinically stable patients without high-risk features, many experts favor aspirin monotherapy, whereas others may consider short-term DAPT or extrapolate acute coronary syndrome guidelines in select cases [1]. In our patient, aspirin monotherapy was chosen given clinical stability, preserved left ventricular systolic function, absence of ongoing ischemia, and low thrombotic risk, aligning with contemporary expert practice for conservative SCAD management. The use of statins in SCAD remains controversial, as current evidence does not support a clear benefit. Beta-blockers, on the other hand, are recommended, supported by a 2017 prospective study involving 327 SCAD patients, which demonstrated an association with reduced recurrence rates [7]. There are currently no formal recommendations regarding inpatient or long-term use of antianginal therapy following a SCAD diagnosis. However, such therapies may be considered in patients with evidence of coronary vasospasm or microvascular dysfunction. For patients who are managed conservatively, AHA recommends inpatient monitoring for 3-5 days [1].

Coronary artery bypass grafting (CABG) or percutaneous coronary intervention (PCI) may be considered in patients with ongoing ischemia despite optimal medical therapy, hemodynamic instability, left main dissection, or involvement of the proximal segments of major epicardial vessels [6]. Also, the presence of intramural hematoma or additional dissection should be considered for revascularization [5]. Revascularization in SCAD poses several challenges, including the risk of dissection extension, iatrogenic dissection, intramural hematoma formation, and stent under-expansion [5]. CABG should be considered in stable patients with either left main involvement or severe proximal 2-vessel dissection [1].

Cardiac rehabilitation is recommended for all patients with SCAD [5]. Although formal guidelines defining specific long-term physical activity restrictions are lacking, available evidence supports participation in structured rehabilitation programs. Current expert recommendations favor regular moderate-intensity aerobic exercise for approximately 30-40 minutes on most days of the week [8]. Light-to-moderate resistance training may also be incorporated, with careful attention to proper breathing techniques and avoidance of the Valsalva maneuver, heavy lifting, and excessive straining. Suggested upper weight limits during resistance training are approximately 9 kg for women and 23 kg for men [8]. Overall, individualized, supervised cardiac rehabilitation provides a safe framework for gradually resuming physical activity while minimizing the risk of recurrence.

Routine follow-up with invasive coronary angiography is not advised, given the risk of provoking further intimal injury if a flap persists. Most SCAD lesions heal spontaneously within 30 days [1]. However, recurrence has been reported in 11-19% of cases [9], recurrent MI secondary to SCAD in 15-22%, and long-term major adverse cardiac events in 15-37% of cases [1]. In our case, the patient made a full recovery and reported no recurrence of chest pain during outpatient follow-up.

## Conclusions

This case underscores the diagnostic challenges associated with spontaneous coronary artery dissection, particularly in male patients with atypical presentations, and highlights the importance of recognizing its characteristic angiographic patterns. Early identification is critical, as management strategies differ substantially from those used for obstructive coronary artery disease. In this patient, conservative medical therapy resulted in symptom resolution and a favorable outcome, reinforcing its role as the preferred approach in clinically stable cases. Increased awareness and timely diagnosis are essential to prevent unnecessary interventions and optimize patient outcomes, while ongoing research is needed to further clarify risk factors and inform evidence-based management of this increasingly recognized condition.
